# Functional Roles of the Charged Residues of the C- and M-Gates in the Yeast Mitochondrial NAD^+^ Transporter Ndt1p

**DOI:** 10.3390/ijms252413557

**Published:** 2024-12-18

**Authors:** Daniela Valeria Miniero, Ferdinando Palmieri, Virginia Quadrotta, Fabio Polticelli, Luigi Palmieri, Magnus Monné

**Affiliations:** 1 Department of Biosciences, Biotechnology and Environment, University of Bari Aldo Moro, Via E. Orabona 4, 70125 Bari, Italy; danielavaleria.miniero@uniba.it (D.V.M.); ferdpalmieri@gmail.com (F.P.); luigi.palmieri@uniba.it (L.P.); 2 Department of Medicine and Surgery, LUM University Giuseppe Degennaro, 70010 Casamassima, Italy; 3 CNR Institute of Biomembranes, Bioenergetics and Molecular Biotechnologies (IBIOM), 70126 Bari, Italy; 4 Department of Sciences, University Roma Tre, Viale G. Marconi 446, 00146 Rome, Italy; virginia.quadrotta@uniroma3.it (V.Q.); fabio.polticelli@uniroma3.it (F.P.); 5 Department of Health Sciences, University of Basilicata, Via Ateneo Lucano 10, 85100 Potenza, Italy

**Keywords:** mitochondrial carrier, mitochondria, mitochondrial transporter, membrane transport, membrane transport protein, NAD^+^ carrier, transport mechanism

## Abstract

Mitochondrial carriers transport organic acids, amino acids, nucleotides and cofactors across the mitochondrial inner membrane. These transporters consist of a three-fold symmetric bundle of six transmembrane α-helices that encircle a pore with a central substrate binding site, whose alternating access is controlled by a cytoplasmic and a matrix gate (C- and M-gates). The C- and M-gates close by forming two different salt-bridge networks involving the conserved motifs [YF][DE]XX[KR] on the even-numbered and PX[DE]XX[KR] on the odd-numbered transmembrane α-helices, respectively. We have investigated the effects on transport of mutating the C-gate charged residues of the yeast NAD^+^ transporter Ndt1p and performed molecular docking with NAD^+^ and other substrates into structural models of Ndt1p. Double-cysteine substitutions and swapping the positions of the C-gate charged-pair residues showed that all of them contribute to the high transport rate of wild-type Ndt1p, although no single salt bridge is essential for activity. The in silico docking results strongly suggest that both the C-gate motif mutations and our previously reported M-gate mutations affect gate closing, whereas those of the M-gate also affect substrate binding, which is further supported by molecular dynamics. In particular, NAD^+^ most likely interferes with the cation-π interaction between R303-W198, which has been proposed to exist in the Ndt1p M-gate in the place of one of the salt bridges. These findings contribute to understanding the roles of the charged C- and M-gate residues in the transport mechanism of Ndt1p.

## 1. Introduction

Mitochondrial carriers (MCs) constitute a protein superfamily of transporters (named the solute carrier family 25 (SLC25) in higher animals) with 53 members in *Homo sapiens*, 35 in *Saccharomyces cerevisiae* and 58 in *Arabidopsis thaliana*, most of them localized in the inner membrane of mitochondria [1,2]. The protein sequences of MCs consist of three repeats, each containing two hydrophobic segments linked by a signature motif sequence PX[DE]XX[KR]X[KR]X_20-30_[DE]GXXXX[WYF][KR]G (PROSITE PS50920, PFAM PF00153 and IPR018108) [2,3]. To determine the substrates transported by MCs, many of their genes have been cloned, and the proteins were expressed recombinantly, purified, reconstituted into liposomes and used in transport assays with radioactive substrates (the EPRA approach) [2,4]. By applying the EPRA method, MCs have been shown to transport various nucleotides, amino acids, metabolites, cofactors and inorganic ions specifically, and the transport properties (kinetic parameters and uniport/antiport/symport modes of transport) of wild-type proteins and human pathogenic variants have been investigated [1,2,5].

MCs have a six-transmembrane segment topology with the N- and C-termini located in the intermembrane space [1,2]. Atomic structures of the ADP/ATP carrier (AAC), a MC superfamily member, demonstrate that the hydrophobic segments form a bundle of six transmembrane α-helices (H1–H6) surrounding a central substrate translocation pore [6,7,8]. Two different conformations of the AAC have been crystallized: one with the inhibitor carboxyatractyloside bound in the central translocation pore, which corresponds approximately to the C-state (the pore is open to the cytoplasmic side and closed to the matrix side by the M-gate) [6,7], and one with the inhibitor bongkrekic acid bound in the pore, which resembles the M-state (the pore open to the matrix and closed to the cytoplasmic side by the C-gate) [8]. The closed M-gate is formed by a proline-induced kink and a salt-bridge network involving the PX[DE]XX[KR] residues of the three signature motifs located towards the matrix of the odd-numbered α-helices [6,7,9,10]. The closed C-gate is formed by a salt-bridge network of the less conserved [YF][DE]XX[KR] motifs located in the vicinity of the cytoplasmic side of the even-numbered α-helices [8,11,12]. In the substrate translocation pore between the two gates, a substrate binding site is formed mainly, but not exclusively, by the protruding residues of the three so-called contact points, which are numbered I, II and III on H2, H4 and H6, respectively [13].

In general agreement with the original “single-binding center gated pore” hypothesis [14], a more detailed common transport mechanism for MCs has been developed based on the structures and on the characterized transport properties, which suggests that most MCs are monomeric antiporters operating with ping-pong kinetics [2,15,16,17,18,19,20]. In the transport cycle, a substrate in the intermembrane space enters the MC in the C-state, binds the centrally-located substrate binding site, which induces conformational changes leading to the closure of the C-gate and opening of the M-gate and exits into the matrix from the MC in the M-state. In an antiport mode of transport, the reverse direction and order of events re-localize another substrate molecule from the matrix to the intermembrane space to complete the transport cycle. In other words, the alternating access of substrates to the translocation pore from the two sides of the membrane is obtained through the C- and M-state conformations and the transition between these states is driven by the energy of the substrate binding to the contact point residues and other residues of the substrate binding site [2,11,13,16,18,21]. This transport mechanism, therefore, requires the salt-bridge networks of the C- and M-gates to form for closing and to break up for opening.

In this study, we have investigated the effects on transport of mutating the charged residues of the [YF][DE]XX[KR] motifs in the *S. cerevisiae* mitochondrial NAD^+^ transporter Ndt1p, which form a complete network of three salt bridges in the C-gate. The Ndt1p mutations (single- and double-cysteine replacements and double, quadruple and sextuple salt-bridge pair swaps) were introduced by site-directed mutagenesis, and the mutant transport activities were assessed by the EPRA method. All the mutations of the charged residues in the Ndt1p [YF][DE]XX[KR] motifs caused a decrease of the wild-type Ndt1p NAD^+^ transport rate, but none of the salt bridges was found to be essential for transport. Structural models of Ndt1p with docking of NAD^+^ and other substrates suggest that the charged residues in the [YF][DE]XX[KR] motifs play an important role in the optimal transport rate by being involved in C-gate opening and closing but not in substrate binding. In addition, our previously published mutagenesis data of the charged residue positions in the Ndt1p M-gate PX[DE]XX[KR] motifs [10] were analyzed in the light of the new docking and molecular dynamics (MD) studies, suggesting that these residues (besides contributing to the M-gate formation) are involved in direct interactions with the substrates in the binding site.

## 2. Results

### 2.1. Effects of Cysteine Substitutions of the Charged Residues in the C-Gate on the Transport Activity of Ndt1p

**Figure 1:**
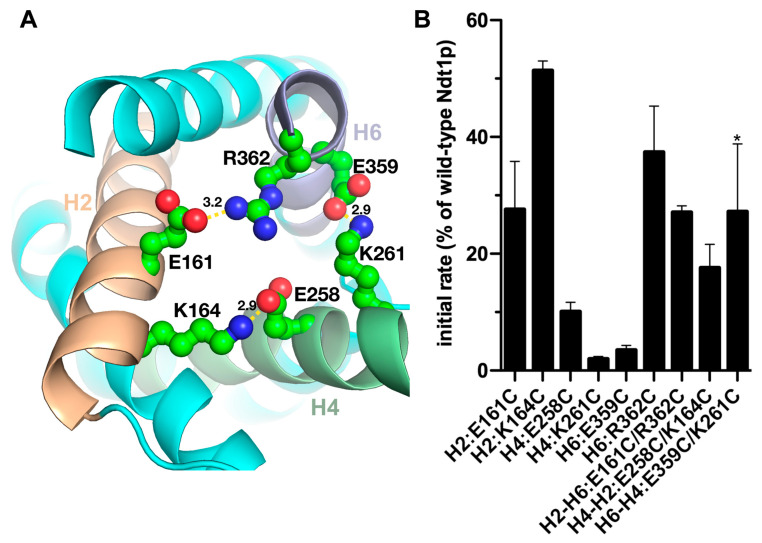
Initial transport rates of the Ndt1p C-gate cysteine mutants. (**A**) The charged residues of the [YF][DE]XX[KR] motifs, which form the salt-bridge network of the closed C-gate in the homology model of Ndt1p in the M-state, are shown from the cytoplasmic (intermembrane space) side of the mitochondrial inner membrane with distances in Å. (**B**) Initial transport rates of Ndt1p cysteine mutant proteins reconstituted in liposomes. Transport was started by adding 3 mM of [^14^C]-NAD^+^ to proteoliposomes containing 20 mM NAD^+^ and stopped after 30 s by the stop inhibitor method. The activity is expressed as the percentage initial rate of wild-type Ndt1p, which was 1183 (±205) μmol/min/g protein. The mean transport rates ± SE displayed were calculated based on the percentage of the wild-type Ndt1p rate in at least three independent experiments carried out in duplicate. The differences in transport activities between the Ndt1p wild-type and all the mutants, as well as the higher activity of the double mutant (indicated with a star) compared to both the corresponding single mutants, are all significant (χ^2^-test *p* < 0.05). * *p* < 0.05.

The charged residues of the [YF][DE]XX[KR] motifs form three salt bridges of the closed C-gate in the homology model of Ndt1p in the M-state (Figure 1A), which was generated by Modeller [22]. The six charged residues of the Ndt1p C-gate were substituted for cysteines one by one by site-directed mutagenesis, generating six single mutant constructs. The constructs were expressed in *Escherichia coli*, and the proteins were purified and reconstituted in liposomes for transport assays following the previously optimized protocols of the EPRA method for this transporter, which has been shown to be an antiporter [10,23]. Initial NAD^+^/NAD^+^ transport rates were determined by measuring [^14^C]-NAD^+^ uptake into the liposomes reconstituted with Ndt1p mutants, and the percentage activity was calculated with respect to that of wild-type Ndt1p (Figure 1B). The transport activities of all the single-cysteine replacement Ndt1p mutants were significantly lower than the wild-type activity. The Ndt1p mutants H4:K261C and H6:E359C were almost totally inactive. In contrast, the other single mutants displayed reduced transport activities to various extents: H2:E161C exhibited 27% activity, H2:K164C 52%, H4:E258C 10% and H6:R362C 37% compared to wild-type Ndt1p. An initial interpretation of these results could be that all the charged residues of the C-gate network are important for transport to different extents: H4:K261 and H6:E359, which form one of the salt bridges of the closed C-gate, appear to be essential for activity, while the others contribute to the high transport rate of wild-type Ndt1p but are not strictly essential. Given the role of these residues in the C-gate salt-bridge network (Figure 1A), the results could imply that reduced transport rates of the mutations are caused by effects on the energy and speed of C-gate closure.

Next, the transport activities of double-cysteine substitution mutations of the salt-bridge charged pairs of the Ndt1p C-gate were assessed to investigate if the salt bridges are important for NAD^+^ transport. All three charged-pair double mutants displayed significantly reduced transport activity compared to that of wild-type Ndt1p: H2-H6:E161C/R362C 27%, H4-H2:E258C/K164C 18% and H6-H4:E359C/K261C 27% (Figure 1B). Interestingly, the double-cysteine mutant of the C-gate salt-bridge H6-H4:E359C/K261C displayed significantly higher activity (27%) compared to those of both the corresponding single-cysteine mutants (below 5%). The comparison between the single and double mutants of this charge pair suggests that H4:K261 and H6:E359 are not essential for transport, in contrast to the initial interpretation reported in the previous paragraph. Furthermore, the fact that the transporter is completely inactivated when one of these charges is left unpaired is indicative of residues of salt bridges that rearrange upon substrate binding [10,24]. Taken together, these results suggest that each of the three C-gate salt bridges contributes to the transport rate of wild-type Ndt1p but that none of them is essential for transport. With reference to the M-state Ndt1p structural model (Figure 1A), these conclusions could mean that the C-gate may still close, but more slowly, when lacking one of the salt bridges and that each salt bridge contributes almost equally to the C-gate closure.

### 2.2. Effects of Swapping the Charged Residue Pairs of the C-Gate on the Transport Activity of Ndt1p

**Figure 2:**
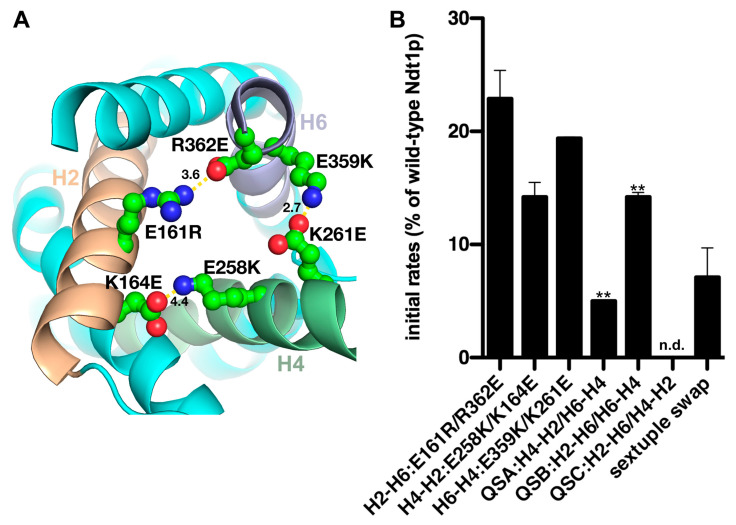
Initial transport rates of the Ndt1p C-gate swapped charged-pair mutants. (**A**) All the charged residues of the [YF][DE]XX[KR] motifs engaged in salt bridges of the closed C-gate (M-state) in the homology model of Ndt1p had their charged pairs swapped by combinations of the mutations indicated (when all of them were substituted as in the sextuple mutation). The protein is shown from the cytoplasmic side of the mitochondrial inner membrane with distances in Å. (**B**) Initial transport rates of Ndt1p swapped charged-pair mutant proteins reconstituted in liposomes. Transport was started by adding 3 mM of [^14^C]-NAD^+^ to proteoliposomes containing 20 mM NAD^+^ and stopped after 30 s by the stop inhibitor method. The activity is expressed as the percentage initial rate of wild-type Ndt1p, which was 1183 (±205) μmol/min/g protein. The mean transport rates ± SE displayed were calculated based on the percentage of the wild-type Ndt1p rate in at least three independent experiments carried out in duplicate. The transport rate of the QSC mutant was not determined (n. d.). The differences in transport activities between Ndt1p wild-type and all the mutants, as well as the lower activity of the two quadruple mutants (indicated with two stars) compared to both their corresponding double-swap mutants, are all significant (χ^2^-test *p* < 0.05). ** *p* < 0.01.

To further investigate the contribution of the C-gate salt bridges to the transport activity of Ndt1p, double mutants with swapped positions of the residues in the charged pairs were made (all possible swaps are shown in Figure 2A), and the mutant proteins were assayed for transport. The three double-charged swapped mutants showed significantly lower transport activities compared to the Ndt1p wild-type level: H2-H6:E161R/R362E 23%, H4-H2:E258K/K164E 14% and H6-H4:E359K/K261E 19% (Figure 2B). It is noteworthy that these transport rates (14–23%) are not significantly different from those of their corresponding double-cysteine mutants (18–27%) (Figure 1B). In conclusion, the position of the negatively and positively charged residues in each salt-bridge pair may not be swapped without a considerable decrease in the transport rates.

Constructs with combinations of swapped charged-pair residues of the Ndt1p C-gate were generated and studied, all exhibiting significantly lower transport activities than that of wild-type Ndt1p. The quadruple-swap mutants QSA (containing H4-H2:E258K/K164E and H6-H4:E359K/K261E) displayed about 5% activity and QSB (H2-H6:E161R/R362E and H6-H4:E359K/K261E) 15%, whereas the QSC (H2-H6:E161R/R362E and H4-H2:E258K/K164E) could not be expressed for unknown reasons (Figure 2B). Thus, the transport rates of the QSA and QSB mutants were significantly lower compared to those of their two corresponding swapped charge-pair double mutants (Figure 2B). Moreover, a sextuple-swap mutant (containing all the three charged residue pairs of the C-gate swapped, as displayed in Figure 2A, i.e., H2-H6:E161R/R362E, H4-H2:E258K/K164E and H6-H4:E359K/K261E) showed about 7% activity of that of wild-type Ndt1p. Taken together, the results of the multiple charged-pair mutants suggest that swapping the positions of the charged pairs does not rescue activity, i.e., the order of the charged residue positions in the salt-bridge network of the C-gate (Figure 1A and Figure 2A) is important for the energy and kinetics of C-gate closing.

### 2.3. Molecular Docking of NAD^+^ into the C-State of Ndt1p

**Figure 3:**
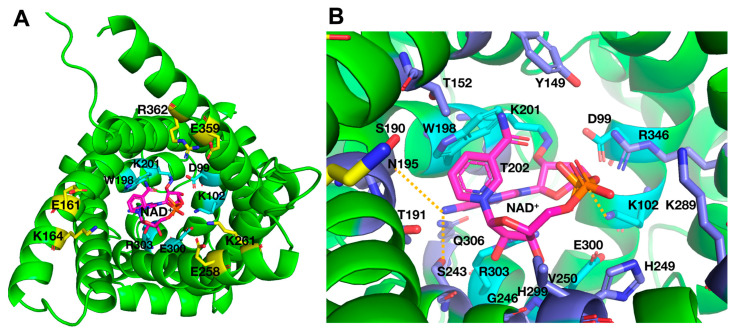
Docking of NAD^+^ into the structural model of Ndt1p by using AlphaFold 3. The top solution of the docking of NAD^+^ in the structural model generated by AlphaFold 3 represents the protein in the C-state. (**A**) Docking solution displayed from the cytosolic side with the charged residue network residues of the open C-gate in sticks with yellow carbons, residues of the closed M-gate in sticks with cyan carbons and NAD^+^ with the carbons in magenta. (**B**) A closer view of the NAD^+^ binding site with all residues having substrate interactions indicated the M-gate residues (sticks with cyan carbons) and other residues (sticks with blue carbons). Hydrogen bonds are indicated by dashed yellow lines. The adenyl moiety of NAD^+^, seen from the lateral side, is intercalated between the M-gate residues W198 and R303, interrupting the presumed cation-π interaction.

The possible involvement of the charged residues of the C-gate [YF][DE]XX[KR] motifs and of the previously investigated M-gate PX[DE]XX[KR] motifs [10] in substrate binding, besides in gate closure, was assessed through molecular docking of NAD^+^ into Ndt1p by using the artificial intelligence-based AlphaFold 3 server [25]. AlphaFold 3 does not allow choosing the conformation of the carrier, and all five resulting docking solutions contained Ndt1p in a conformation very similar to the C-state structures of AAC with carboxyatractyloside, in which the C-gate is open and the M-gate is closed [6,7]. The results show that NAD^+^ did not interact with any of the C-gate residues in any of the docking solutions (Supplementary Figure S1A,B). However, in all docking solutions, the adenyl moiety of NAD^+^ intercalated between W198 and R303 of the charged network of the M-gate PX[DE]XX[KR] motifs (W198 in the position of [DE]), whereas the rest of the substrate molecule extended towards the cytoplasmic side of the transporter cavity with slightly different conformations in the different solutions (Supplementary Figure S2A). In the top-ranked solution, for example, NAD^+^ interacted with all of the charged network residues of the M-gate (Figure 3A), which have previously been shown to be important for the transport rate [10], and all the residues indicated in Figure 3B, among them, residues belonging to the contact points I (Y149 and T152), II (G246) and III (R346), which have been suggested to take part in substrate binding [13]. In the same top-ranked solution, the interactions of the adenyl base of NAD^+^ with W198 and R303 are compatible with π-π and cation-π interactions, respectively (Supplementary Figure S2B). In the absence of the substrate, W198 and R303 may form a cation-π interaction between themselves, as shown by a structural model of Ndt1p without NAD^+^ docking that was generated by AlphaFold 3 (Supplementary Figure S2C). In addition, other Ndt1p substrates, such as AMP, ADP, ATP, GDP, GTP and FAD, were tested with the same docking approach as with NAD^+^. In all the resulting docking solutions, Ndt1p was in the C-state conformation, and the purine moieties of the different substrates (adenyl for all of them except guanyl in the case of GDP and GTP) were always located between W198 and R303 (the top docking solution of each ligand is shown in Supplementary Figure S3). The rest of the substrate molecules expanded towards the open cytoplasmic side of the cavity with slightly different conformations in the different solutions. Notably, none of the substrates interacted with the C-gate residues. In summary, these docking results suggest that in the C-state conformation, NAD^+^ and other Ndt1p substrates do not interact with the C-gate residues but with residues of the M-gate (especially W198 and R303), besides the contact points and other residues in their vicinity.

### 2.4. MD Simulations of the NAD^+^ Interaction with Ndt1p in the C-State

MD simulations provided valuable insights into the behavior of the C-state Ndt1p-NAD⁺ complex predicted by AlphaFold 3, with particular reference to the most relevant protein–ligand interactions. As outlined in the Methods section, three 200 ns replicas of the system were simulated.

**Figure 4:**
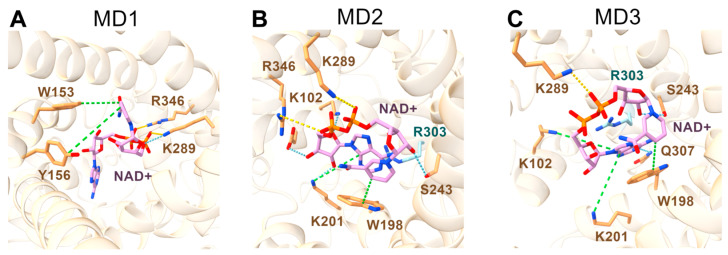
Representative snapshots of the Ndt1p-NAD^+^ complex in the three MD replicas. Snapshots from the MD simulations MD1, MD2 and MD3 are displayed in (**A**–**C**), respectively. Hydrogen bonds are shown as light blue dashed lines, salt bridges as yellow dashed lines and hydrophobic and cation-π interactions as green dashed lines. The protein is represented as ribbons, in beige, while the interacting residues are shown as sticks, colored in orange; the NAD^+^ molecule is represented as sticks, colored in plum. R303, mentioned in the text but not involved in direct interactions with the ligand, is shown as sticks and colored in light blue.

During the first simulation (MD1), NAD⁺ established a stable salt bridge with R346, persisting for over 80% of the trajectory, and a less stable interaction with K289, present for approximately 50% of the simulation trajectory (Figure 4A, Supplementary Figure S4 and Table S1). It must be noted that R346 is in contact point III, which is a highly conserved arginine residue throughout most of the MC superfamily [13]. Additionally, K289 formed a hydrogen bond with NAD⁺, observed for 18% of the trajectory. Hydrophobic interactions between NAD⁺ and W153 and Y156 (which are in contact point I) were also detected, though less persistent, lasting 13.2% and 11.1% of the simulation time, respectively. Notably, W153 also engaged in a cation-π interaction with the positively charged nitrogen of NAD⁺, with a duration of 30.9%. This result is in agreement with the docking simulations. It is important to note that MD1 captured a conformational change event involving NAD⁺. In fact, in the initial AlphaFold 3 structure, NAD⁺ adopted a closed conformation, with the nicotinamide and adenine rings nearly forming a π-π stacking interaction with each other. Approximately 15 ns into the simulation, the ligand underwent an opening event. By around 60 ns, NAD⁺ closed again, although in a different orientation with respect to the starting pose.

In contrast with MD1, throughout MD2 and MD3, NAD⁺ maintained its initial pose, demonstrating a more stable interaction network (Figure 4B,C and Supplementary Figures S5 and S6 and Tables S2 and S3). In detail, during MD2, NAD⁺ formed a strong, persistent hydrogen bond with D99 of the M-gate, which endured for almost the entire simulation. A hydrogen bond with S243 was also observed in both MD2 and MD3, with durations of 57.5% and 35.8% of the respective trajectories. Additional hydrogen bonds were identified in MD2 with the M-gate residues K102 and K201, persisting for 27% and 16.2% of the trajectory, respectively. Interestingly, K201 also engaged in a highly stable cation-π interaction with the adenyl moiety of NAD⁺, observed for 92% of MD2 and 71.5% of MD3. NAD⁺ consistently formed also a salt bridge with K289 in both MD2 and MD3, persisting for 61.8% and 81.2% of the trajectories, respectively. In MD3 alone, and as observed in MD1, NAD⁺ also established a salt bridge with R346 (contact point III) for approximately half of the simulation time. Regarding hydrophobic contacts, NAD⁺ interacted with W198 (M-gate) for 82.3% of MD2 and 51.3% of MD3 (Supplementary Figures S5 and S6). This interaction alternated between a classic 4 Å hydrophobic contact and a cation-π interaction, where the charged nitrogen of NAD⁺ served as the acceptor, and the indole group of W198 acted as the donor, confirming once again the docking simulations results. Additionally, a second cation-π interaction with K102 was exclusive of MD3, persisting for 34.9% of the trajectory. Another interaction exclusive of MD3 was the one involving Q307, which formed a hydrogen bond with NAD^+^ for 22.1% of the simulation time.

Interestingly, NAD⁺ did not establish stable interactions with any of the C-gate residues across the three replicas. In contrast, during MD2 and MD3, it formed multiple stable interactions with four of the six M-gate residues, in particular, D99, K102, W198 and K201. Notably, during MD2 and MD3, the adenyl moiety of NAD⁺ intercalated between W198 and R303 within the charged network of the M-gate PX[DE]XX[KR] motifs, with W198 occupying the [DE] position. These results are largely in agreement and confirm the hypotheses drawn from the docking data (see above), especially the potential interaction between NAD⁺ and W198, whereas a direct bond between NAD⁺ and R303 was not observed.

### 2.5. Molecular Docking of NAD^+^ into the M-State of Ndt1p

**Figure 5:**
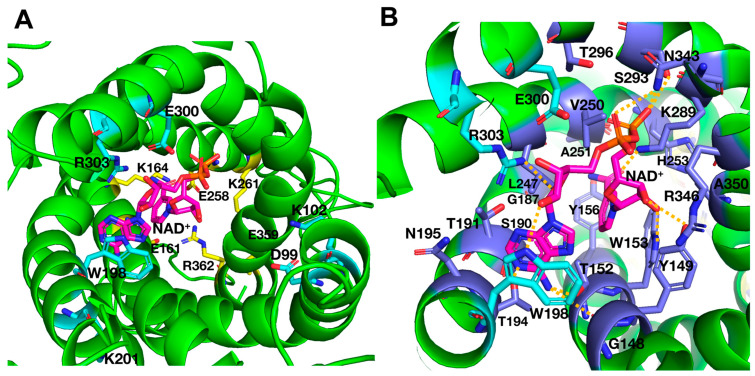
Docking of NAD^+^ into the structural homology model of Ndt1p in the M-state by using AutoDock Vina. The fifth top solution of the docking of NAD^+^ in the structural homology model in the M-state generated by AutoDock Vina. (**A**) The docking solution displayed from the matrix side with the charged residue network residues of the open M-gate in sticks with cyan carbons, residues of the closed C-gate in sticks with yellow carbons and NAD^+^ with the carbons in magenta. (**B**) A closer view of the NAD^+^ binding site with all residues having substrate interactions indicated the M-gate residues W198, E300 and R303 (sticks with cyan carbons) and other residues (sticks with blue carbons). Hydrogen bonds are indicated by dashed yellow lines. The adenyl moiety of NAD^+^ is intercalated between the M-gate residues W198 and R303.

Since the docking solutions of Ndt1p with AlphaFold 3 were all in the C-state (described in two sections previously), we used AutoDock Vina v1.2.x [26] for the docking of NAD^+^ and other substrates into the substrate translocation cavity of the homology model of Ndt1p in the M-state to evaluate potential substrate interactions with the C- and M-gate residues. All the nine docking solutions resulting from AutoDock Vina exhibited very similar NAD^+^ binding energies of between −10.7 to −10.1 kcal/mol, on which a ranking is based. In none of these docking solutions did NAD^+^ interact with any of the charged C-gate residues, which were at least 12 Å away from the substrate, with many other residues of the closed C-gate in between (Supplementary Figure S1C,D). In solution number five (NAD^+^ binding energy of −10.3 kcal/mol), which was the most similar docking solution to those obtained with the Ndt1p C-state by AlphaFold 3, the NAD^+^ adenyl moiety was intercalating between W198 and R303 of the open M-gate, while the rest of the molecule extended towards the cytoplasmic side of the transporter cavity (Figure 5A). Furthermore, all the residues indicated in Figure 5B interacted with NAD^+^; among them, almost all those of the contact points: I (Y149, T152, W153 and Y156), II (L247) and III (R346). Although the position, conformation and interacting residues of NAD^+^ were similar in the docking solutions in the C- and M-states of Ndt1p, the molecular details were very different, i.e., the atoms of the substrate and protein that participated in the interactions. These differences might reflect the possibility that the substrate binding site is in two distinct conformations in the C- and M-states of the carrier, as well as differences in the two docking approaches. Therefore, we refrain from drawing other conclusions from the M-state docking than some general cautious ones that are in support of the C-state docking results, i.e., NAD^+^ lacks interactions with the charged residues of the C-gate, and it is positioned with the adenyl moiety between W198 and R303.

## 3. Discussion

In this study, the effects of mutating the charged residues of the C-gate [YF][DE]XX[KR] motifs on the transport activity of Ndt1p were investigated. The results (Figure 1 and Figure 2) demonstrate that (i) none of the three salt bridges of the C-gate is essential for function, but (ii) they all contribute to the transport rate and (iii) the pairing of some of the charged residues and their order around the network are important for the Ndt1p transport rate. These conclusions are probably valid for all MCs, as strengthened by several lines of evidence. The C-gate [YF][DE]XX[KR] motifs, which are less conserved than the M-gate PX[DE]XX[KR] motifs, often form an incomplete charged-pair network in other MCs [11]. This observation by itself implies that not all three salt bridges are essential for transport function in all MCs. Furthermore, few of the single mutations of the C-gate charged-pair residues in other MCs completely diminished the carrier activity: the only charged pair in bOGC (H6-H4) E301C/A/D/G (<10% of wild-type activity) and K206C (>50%) [27,28]; the two in pMir1p (H2-H6) E95Q (>60%) and K295A (>30%), and (H4-H2) E192Q (>30%) and K98A (<15%) [29,30,31]; the two in CAC (H4-H2) E191A (>50%) and K97A (>50%), and (H6-H4) E288A (>30%) and K194A (>50%) [32]. Combinations of mutations have been made not only in Ndt1p (this study) but also in yeast Aac2p, which has an incomplete network (a glutamine instead of a negatively charged residue in the third motif): when all network residues of Aac2p were mutated into negatively or positively charged residues, transport was virtually abolished, whereas the mutant with the position of all the charged-pair residues inverted displayed 14% activity [7]. It is noteworthy, that none of these other mutated MCs have a complete network of three salt bridges in the C-gate as Ndt1p. Thus, the “mild” conservation of the C-gate [YF][DE]XX[KR] motifs and the transport activities of several mutants in other MCs support the three general conclusions (i–iii above) drawn from the results of this study.

In the structural docking models, the charged residues of the C-gate are not in contact with NAD^+^ bound in the Ndt1p substrate binding site, neither in the C-state nor in the M-state (Supplementary Figure S1). This conclusion is also supported by the results of the MD simulations of the C-state complex. It is likely that this is the case for all substrates of all MCs; this conclusion can also be deduced from the AAC M-state structure, in which there are many residues (among them [YF] of the [YF][DE]XX[KR] motifs) forming a 15 Å “hydrophobic plug” between the substrate binding pocket and the charged C-gate residues [8,18]. Considering the conclusions i–iii, our results may be interpreted to indicate that the alternation of negatively and positively charged residues in a circle around the whole C-gate salt-bridge network is important for achieving the wild-type Ndt1p transport rate level. This interpretation is in favor of the idea of the charge relay switch [33] in which intrahelical salt bridges are formed between the charged residues of each [YF][DE]XX[KR] motif, at least temporarily, during the opening of the gate, in contrast to the interhelical ones between the three motifs when the gate is closed. However, the C-gate charged network of Ndt1p could not be inverted completely without almost total loss of transport (the sextuple mutant), and two of the residues of the charged pairs could not be left unpaired. Therefore, it cannot be excluded that the position of some specific C-gate residues may also be important for interactions with other surrounding residues or with the substrate during substrate translocation pore entry or exit on the cytoplasmic side. Taken together, the charged residues of the C-gate are most likely important mainly for forming the salt bridges of the closed C-gate. Thus, the reduced transport activity of the C-gate Ndt1p mutants may be explained by effects on the transport kinetics (by having an impact on the energies involved in opening and closing the C-gate, affecting the speed of entry and exit of the substrate) rather than on direct substrate binding.

The effects on the transport rate of mutating the charged residues of Ndt1p that participate in the salt-bridge network of the closed M-gate were investigated in a previous study [10] and can now be evaluated also with respect to substrate binding through the docking and MD studies presented here. Briefly, the two salt bridges of the Ndt1p M-gate were found to be non-essential for function but important for the transport rate, whereas the cysteine replacements of W198 in the [DE] position of the second PX[DE]XX[KR] motif and R303 in the third motif, singly and together, diminished the transport activity almost completely [10]. This observation led to the proposal that these two residues form a cation-π interaction essential for transport instead of the salt bridge usually present in other MCs in this location [10,13]. In the present study, the docking and MD simulation results suggest that NAD^+^ interacts with M-gate residues, along with the contact points and neighboring residues, in the substrate binding site of Ndt1p (Figure 3, Figure 4 and Figure 5). In particular, the AlphaFold 3 docking and the MD simulations clearly predict that the adenyl moiety of NAD^+^ is positioned between the Ndt1p M-gate residues W198 and R303, that in the absence of substrate, may form a cation-π interaction (Figure 3 and Figure 4 and Supplementary Figures S2, S5 and S6). Moreover, the adenyl or guanyl groups of the many other Ndt1p substrates are also positioned between these two residues (Supplementary Figure S3). This substrate-carrier conformation may also be conserved among the other MCs containing a tryptophan residue corresponding to W198 of Ndt1p (Supplementary Figure S7). As a note of proof, AlphaFold 3 docking of the substrates into some of these other MCs (NAD^+^ in *S. cerevisiae* Ndt2p; NAD^+^ in *A. thaliana* PXN, NDT1 and NDT2; GTP in human SLC25A36; and FAD in human SLC25A32) shows that in all solutions the purine moieties of the substrates are also positioned between this tryptophan and the arginine residues corresponding to R303 of Ndt1p in the C-state of the MCs (Supplementary Figure S8), with the exception of PXN (which is the only one that has a lysine instead of an arginine). Although one should be cautious with ligand docking predictions, and some of the atomic details may be incorrect, it is likely that the suggested purine sandwich W198-NAD^+^-R303, of which the W198-NAD^+^ interaction is also supported by the MD simulations, is indeed occurring because the same basic result was obtained in all solutions by both varying the proteins and the substrates. In addition, there are many structures of ATP-binding proteins, which are not MCs, that bind the adenine base with arginine/lysine and phenylalanine/tyrosine/tryptophan residues through cation-π and π-π interactions, respectively [34]. Therefore, we propose that the NAD^+^ binding in Ndt1p interferes with the W198-R303 interaction, which would lead to a change in the matrix network strength and subsequent M-gate opening/closing. Notably, this key-into-lock-like mechanism is conserved among nearly all the MC subfamilies that transport NAD^+^, FAD, coenzyme A and pyrimidine nucleotides.

Our findings that all three salt bridges of the C-gate and the two of the M-gate of Ndt1p contribute to an optimal transport rate without being essential for transport as well as the substrate docking and MD predictions (Figure 3, Figure 4 and Figure 5 and Supplementary Figures S1–S8) are all compatible with the previously proposed model for the conformational changes of the substrate translocation mechanism of AAC (and all MCs) [8,18,35]. This mechanism involves six mobile elements: binding of the substrate to the contact point residues found at hinge regions between the three “gate elements” (the C-terminal part of the even-numbered α-helices containing the C-gate [YF][DE]XX[KR] motifs) and the three “core elements” (the odd-numbered α-helices containing the M-gate PX[DE]XX[KR] motifs, the matrix helices and the N-terminal part of the even-numbered α-helices) induces the alternation between the M- and the C-states. Therefore, the strengths of the C- and M-gate salt-bridge networks areoptimized with respect to the binding energy of the substrate. The only difference with the proposed transport mechanism [18] is that we suggest that NAD^+^, bound in the substrate binding site in the central cavity of Ndt1p, interacts directly with M-gate residues, besides the residues of the three contact points and additional residues in their vicinity. In addition to the supporting results for these interactions, it also seems logical and likely that large MC substrates interact both with the contact points and M-gate residues because the central cavity is narrow, and these two groups of residues are closely spaced. Therefore, direct NAD^+^ interactions with residues of the contact points and M-gate may trigger the opening and closing of the M-gate, and these conformational changes may be propagated and lead to the opening and closing of the C-gate. Furthermore, these conclusions offer an explanation for the conserved MC subfamily variations of the C- and M-gate motifs (adjusting the energy requirements of the conformational changes involving gate opening and closing) and for the variations in the M-gate motifs (also for adapting the specific substrate binding).

## 4. Materials and Methods

### 4.1. Materials

[^14^C]-NAD^+^ was purchased from Perkin Elmer, Italy; PIPES, Triton X-114, Amberlite XAD-4 and egg yolk phospholipids (lecithin from eggs) were from Fluka (Milan, Italy); *N*-dodecanoylsarcosine (sarkosyl) was from Sigma (Milan, Italy); and Sephadex G-75 was from Pharmacia (Milan, Italy). All reagents were of analytical grade.

### 4.2. Construction of Plasmids and Site-Directed Mutagenesis

The DNA sequence of wild-type Ndt1p was used as a template to generate single- and double-cysteine replacement and inverted charged-pair mutants, as described previously (the primers are in Supplementary Figure S9) [10]. All mutations were introduced by the overlap extension PCR method [36] using oligonucleotides with appropriate mutations in their sequences. The PCR products were cloned into the expression vector pMW7 and transformed into *E. coli* DH5α cells. Transformants, which were selected on 2 × TY plates containing ampicillin (100 μg/mL), were screened by direct colony PCR and by restriction digestion of the purified plasmid DNA. All mutant constructs were verified by DNA sequencing.

### 4.3. Overexpression and Purification of the Recombinant MC Proteins

The proteins were overproduced as inclusion bodies in the cytosol of *E. coli*, as described previously [10,23]. The inclusion bodies were purified by sucrose layer density gradient centrifugation and washed at 4 °C with TE buffer (10 mM Tris-HCl, pH 8.0 and 1 mM EDTA). Then, they were washed twice with a buffer containing 10 mM PIPES, pH 7.0, 3 % (*w*/*v*) Triton X-114, 1 mM EDTA, and 20 mM Na_2_SO_4_, and finally with TE buffer again. The recombinant proteins were solubilized in a buffer containing 2.5% (*w*/*v*) sarkosyl, 1 mM EDTA and 10 mM Tris-HCl at pH 7.0. The residual material was removed by centrifugation (258,000× *g* for 1 h at 4 °C).

### 4.4. Reconstitution of the Purified MC Proteins into Liposomes and Transport Measurements

The recombinant proteins in sarkosyl were reconstituted into liposomes in the presence of substrate, as described previously [10,23]. The external substrate was removed from the proteoliposomes on Sephadex G-75 columns. Transport at 25 °C was started by adding the radiolabeled substrate to the eluted proteoliposomes and terminated by the addition of 20 mM pyridoxal 5′-phosphate and 16 mM bathophenanthroline, which in combination inhibit the activity of several mitochondrial carriers completely and rapidly. In the controls, the inhibitors were added at the beginning together with the labeled substrate according to the “inhibitor stop” method. Finally, the external radioactivity was removed on Sephadex G-75 columns, and the radioactivity in the proteoliposomes was measured. The experimental values were corrected by subtracting the control values. The initial transport rates were calculated from the radioactivity taken up by the proteoliposomes after 30 s, i.e., in the initial linear range of substrate uptake, and were corrected by taking into account the efficiency of reconstitution, i.e., the proportion of reconstituted protein.

### 4.5. Additional Experimental Methods

SDS-PAGE was performed essentially as described previously [10]. The amount of pure recombinant proteins was estimated from Coomassie Blue-stained SDS-PAGE gels with the Bio-Rad GS-700 Imaging Densitometer (Bio-Rad Laboratories, San Francisco, CA, USA) using carbonic anhydrase as a protein standard. The extent of incorporation of recombinant proteins into liposomes was determined as described, except that the protein concentration was determined by laser densitometry of stained SDS gels after extraction of lipids by organic solvents.

### 4.6. Molecular Docking and Homology Modeling

Molecular docking in Ndt1p in the C-state was performed with AlphaFold 3 [25] by giving the protein sequence of the MC transporter domain of Ndt1p (residues 70-373, excluding the N-terminal extension) and indicating the substrate NAD^+^ or the other possible substrates as ligand. The homology model of Ndt1p in the M-state was generated by using the structure of the bongkrekic acid-inhibited *Thermothelomyces thermophila* AAC [8] as a template with Modeller [22]. Semi-rigid molecular docking of NAD^+^ was performed with AutoDock Vina [26] into the Ndt1p M-state homology model, with the substrate and residues with side chains exposed to the substrate translocation cavity given conformational flexibility: V95, D99, K102, T102, I145, Y149, T152, W153, Y156, W198, K201, T202, S243, L247, V250, H253, K289, S293, T296, Y297, E300, R303, T304, N343 and R346. A grid box with the dimensions 26 × 24 × 28 Å enclosing all flexible residues was used in the docking. Protein–ligand interactions in the docking solutions were analyzed by PISA v1.48 [37].

### 4.7. MD Simulations

The ionizable residues’ protonation state of the top-ranked solution of the C-state Ndt1p-NAD^+^ complex obtained by AlphaFold 3 was predicted using PDB2PQR v3.6.1, with the default PARSE force field, and the implemented PROPKA [38,39]. The protein–ligand complex was then embedded into a phospholipid bilayer mimicking the inner mitochondrial membrane using the CHARMM-GUI web server (http://www.charmm-gui.org; accessed on 27 November 2024) [40,41,42]. The membrane composition was based on the inner mitochondrial membrane model published by the CHARMM-GUI team, available in their archive (https://charmm-gui.org/?doc=archive&lib=biomembrane; accessed on 27 November 2024) [43]. To accurately represent the differences between the inner and outer leaflets of the IMM, adjustments were made to the composition and lipid tail structure. Full details of the membrane composition are provided in Supplementary Table S4. Water molecules, modeled using the TIP3P model, were added to both sides of the membrane, creating two layers, each 22.5 Å thick. NaCl ions were included to neutralize the system and achieve a physiological ionic strength of 0.15 M. The CHARMM36m force field [44] and the AMBER22 package [45] were employed for the MD simulations of the assembled systems, consisting of approximately 100,000 atoms. Simulations followed the standard CHARMM-GUI protocol. Energy minimization was initially performed with 2500 steps of the steepest descent, and positional restraints were applied to the protein residues (10 kcal mol^−1^ Å^−2^) and to the membrane (2.5 kcal mol^−1^ Å^−2^). After minimization, simulations were carried out using the canonical NVT ensemble to raise the temperature to 310.15 K, followed by isothermal-isobaric NPT ensemble simulations to stabilize the pressure at 1 bar. During the thermalization and equilibration phases, the positional restraints were gradually decreased. Once the system reached equilibrium, it was further simulated in three different replicas of 200 ns each without restraints using the NPT ensemble. The Langevin thermostat was used during both the NVT and NPT simulations, while pressure control was managed with a Monte Carlo barostat with semi-isotropic pressure scaling [46]. All simulations were performed under periodic boundary conditions. Long-range non-bonded interactions were treated with the particle mesh Ewald (PME) method [47] using a 12 Å cutoff and a force-switching region beginning at 10 Å. The production runs were performed with a time step of 2 fs. NAD^+^ was treated in the same manner as the protein residues, with positional restraints applied during the initial equilibration phases.

### 4.8. MD Simulation Analyses

All analyses were performed using the CPPTRAJ package [48]. Only the protein–ligand interactions with a persistence above 10% of the simulation time were included to focus on the most significant ones. Salt bridges were analyzed using the *hbond* command, with a cutoff distance of 4 Å and no angle cutoff. For salt bridges, only the charged residues of the protein and the charged atoms of the ligand were considered. Hydrophobic contacts were identified with the *nativecontacts* command. For the cation-π interactions analysis, the distance between the geometric center of the aromatic ring and the positively charged group and the angle formed between the cation and the aromatic ring plane were monitored.

Backbone RMSD was calculated using the *rmsd* command, while RMSF was computed on α-carbons using the *atomicfluct* command. The mass densities for lipid headgroups, glycerol ester groups, acyl chains and solvent were determined with the *density* command. The bilayer membrane thickness was calculated using the MDAnalysis Python library [49,50]. Lastly, raw data were processed and visualized using the Python libraries Pandas and Matplotlib [51,52]. The results of all the above-cited analyses are reported in Supplementary Figures S10–S13.

## Data Availability

The original contributions presented in this study are included in the article/supplementary material. Further inquiries can be directed to the corresponding author.

## Supplementary Materials

The following supporting information can be downloaded at: https://www.mdpi.com/article/10.3390/ijms252413557/s1.

## Abbreviations

AAC, ADP/ATP carrier; C-gate, cytoplasmic gate; C-state, cytoplasmic state; EPRA, expression, purification, reconstitution and transport assay; MC, mitochondrial carrier; MD, molecular dynamics; M-gate, matrix gate; M-state, matrix state; Ndt1p, mitochondrial NAD^+^ transporter.
