# Supplementary material for: Functional Roles of the Charged Residues of the C- and M-Gates in the Yeast Mitochondrial NAD+ Transporter Ndt1p
Source: Int J Mol Sci. 2024 Dec 18;25(24):13557. doi: 10.3390/ijms252413557 (PMC11677788; doi:10.3390/ijms252413557)
Supplement: Supplementary file 1 [file ijms-25-13557-s001.zip › ijms-3326032-supplementary.pdf]

Supplementary material for the article:

Functional roles of the charged residues of the C- and M-gates in the yeast mitochondrial NAD^+ transporter Ndt1p

Authors

Daniela Valeria Miniero[1,2], Ferdinando Palmieri[1,3], Virginia Quadrotta[4], Fabio Polticelli[4], Luigi Palmieri[1,3] and Magnus Monné[1,5,*]

Affiliations

1. Department of Biosciences, Biotechnology and Environment, University of Bari Aldo Moro, Bari, Italy
2. Department of Medicine and Surgery, LUM University Giuseppe Degennaro, Casamassima, Italy
3. CNR Institute of Biomembranes, Bioenergetics and Molecular Biotechnologies (IBIOM), Bari, Italy
4. Department of Sciences, University Roma Tre, Rome, Italy
5. Department of Health Sciences, University of Basilicata, Potenza, Italy

* corresponding author: magnus.monne@unibas.it

Supplementary Figs. S1-S13

Supplementary Table S1-S4

Supplementary Fig. S1. The docking of NAD^+ in the structural models of the C- and M-states of Ndt1p. (**A**) The docking solution in the C-state (also shown in **Fig. 3**) is displayed from the lateral membrane side and slightly cut with the charged residue network residues of the closed M-gate in sticks with cyan carbons, residues of the open C-gate in sticks with yellow carbons and NAD^+ with the carbons in magenta. (**B**) A closer view at the NAD^+ binding site of the C-state model Ndt1p from the cytosolic side with all residues in green spheres, except for the M-gate residues, which are in cyan, and the C-gate residues, which are in yellow. (**C**) The docking solution in the M-state (also shown in **Fig. 5**) is displayed from the lateral membrane side and slightly cut with the charged residue network

residues of the open M-gate in sticks with cyan carbons, residues of the closed C-gate in sticks with yellow carbons and NAD^+ with the carbons in magenta. The closest distance (in Å) between NAD^+ and the C-gate residues is shown. (**D**) A closer view at the NAD^+ binding site of the M-state model Ndt1p from the matrix side with all residues in green spheres, except for the M-gate residues, which are in cyan, and the C-gate residues, which are in yellow. The latter residues can hardly be seen because many other residues of the closed C-gate are between them and NAD^+.

Supplementary Fig. S2. Interactions of W198 and R303 of the M-gate in the Ndt1p C-state conformation model with and without NAD^+. The closest distances are in Å. (**A**) Superimposition of all five NAD^+ docking solutions generated by using AlphaFold 3, showing the adenyl bases (lines in different colors) between W198 and R303 (in overlayed spheres). (**B**) The top-ranked NAD^+ docking solution showing the adenyl moiety between W198 and R303 (in sticks). (**C**) The structural model of Ndt1p made in the same way as the AlphaFold 3 docking but in the absence of NAD^+, which shows that the distance between W198 and R303 is compatible with forming a cation-π interaction.

Supplementary Fig. S3. Docking of various substrates into the structural model of C-state Ndt1p by using AlphaFold 3. The top solutions of the docking of: (**A**) NAD^+ (sticks with green carbons), (**B**) AMP (sticks with cyan carbons), (**C**) ADP (sticks with magenta carbons), (**D**) ATP (sticks with pink carbons), (**E**) GDP (sticks with yellow carbons), (**F**) GTP (sticks with white carbons) and (**G**) FAD (sticks with blue carbons) in the structural model generated by AlphaFold 3 viewed from the cytoplasmic side. The adenyl and guanyl moieties were positioned between W198 and R303 of the M-gate. (**H**) All docking solutions of **A**-**G** superimposed.

Supplementary Fig. S4. Distances between NAD^+ and binding residues during MD1.

Supplementary Fig. S5. Distances between NAD^+ and binding residues during MD2.

Supplementary Fig. S6. Distances between NAD^+ and binding residues during MD3.

Supplementary Fig. S7. Sequence alignment of the M-gate PX[DE]XX[KR] signature motifs of the second and third repeats. The bovine AAC sequence was included as a reference for the alignment of the MC sequences containing a conserved tryptophan (marked in red) instead of a negatively charged residue in the M-gate PX[DE]XX[KR] motif (in bold) of the second repeat; the conserved arginine in the third repeat is marked in cyan. *S. cerevisiae* Ndt1p and Ndt2p transport NAD^+, (d)AMP and (d)GMP; *S. cerevisiae* Rim2p transports pyrimidine nucleotides; *A. thaliana* PXN transports coenzyme A, adenosine 3′, 5′-phosphate, NAD^+, AMP and ADP; *A. thaliana* NDT1 and NDT2 transport NAD^+, ADP and AMP; *H. sapiens* SLC25A17 transports CoA, NAD^+ and FAD; *H. sapiens* SLC25A32 transports FAD and folate; *H. sapiens* SLC25A33 and SLC25A36 transport pyrimidine nucleotides.

Supplementary Fig. S8. Docking of various substrates into structural models of different MCs with a tryptophan instead of a negatively charged residue in signature motif 2 by using AlphaFold 3. Close view of the top docking solutions of NAD^+ (sticks with magenta carbons, **A-D**), GTP (sticks with white carbons, **E**) and FAD (sticks with blue carbons, **F**) in the structural models: *S. cerevisiae* Ndt2p (**A**); *A. thaliana* PXN (**B**) NDT1 (**C**) and NDT2 (**D**); human SLC25A36 (**E**) and SLC25A32 (**F**). The adenyl and guanyl moieties in all solutions (in the C-state) are fixed between the tryptophan in motif 2 and the arginine in motif 3 of the M-gate with the exception of Pxn1p (**B**). Notably, this is the only one of these MCs that has a lysine instead of an arginine in the third motif. In all other docking solutions the position of the substrate was similar to that of the top ranked one as shown here.

Supplementary Fig. S9. Primer sequences used for site-directed mutagenesis of Ndt1p. For the single cysteine mutations the forward (for) and reverse (rev) primer couples were used with the wild-type Ndt1p construct as template. For the double, quadruple and sextuple mutants combinations of primers were used with the already mutated Ndt1p template constructs.

Supplementary Fig. S10. RMSD time series. Plots of the RMSD time series for each simulation, calculated for the protein backbone from residue 1 to residue 305, which correspond to residues 69-373 of the wild-type sequence.

Supplementary Fig. S11. RMSF analysis. Plots of the RMSF for each simulation calculated from residue 1 to 305, which correspond to residues 69-373 of the wild-type sequence.

Supplementary Fig. S12. Mass density calculated for lipids headgroups, glycerol ester groups, acyl chains, and for the solvent, during each simulation.

Supplementary Fig. S13. Bilayer lipid membrane thickness time series. Thickness of the bilayer lipid membrane is plotted against simulation time.

Supplementary Table S1. **Interactions established by NAD^+ during the first MD simulation.** For each pair, only the most frequent interaction is reported and only those with a persistence higher than 10% of the trajectory are shown. For cation-π interactions only the most frequent interaction is reported but the evaluation of the interaction has been carried out considering the distance between the geometric center of the aromatic ring and the positively charged group, as well as the angle formed between the cation and the aromatic ring plane.

Supplementary Table S2. Interactions established by NAD^+ during the second MD simulation. For each pair, only the most frequent interaction is reported and only those with a persistence higher than 10% of the trajectory are shown. For cation-π interactions only the most frequent interaction is reported but the evaluation of the interaction has been carried out considering the distance between the geometric center of the aromatic ring and the positively charged group, as well as the angle formed between the cation and the aromatic ring plane. The abbreviation ADE indicates the adenosine group of NAD^+, whose ring is involved in cation-π interactions.

Supplementary Table S3. Interactions established by NAD^+ during the third MD simulation. For each pair, only the most frequent interaction is reported and only those with a persistence higher than 10% of the trajectory are shown. For cation-π interactions only the most frequent interaction is reported but the evaluation of the interaction has been carried out considering the distance between the geometric center of the aromatic ring and the positively charged group, as well as the angle formed between the cation and the aromatic ring plane. The abbreviation ADE indicates the adenosine group of NAD^+, whose ring is involved in cation-π interactions.

Supplementary Table 4. Membrane composition for MD simulations. During membrane building with CHARMM-GUI the following number of lipids and mean area per lipid (APL) have been selected.

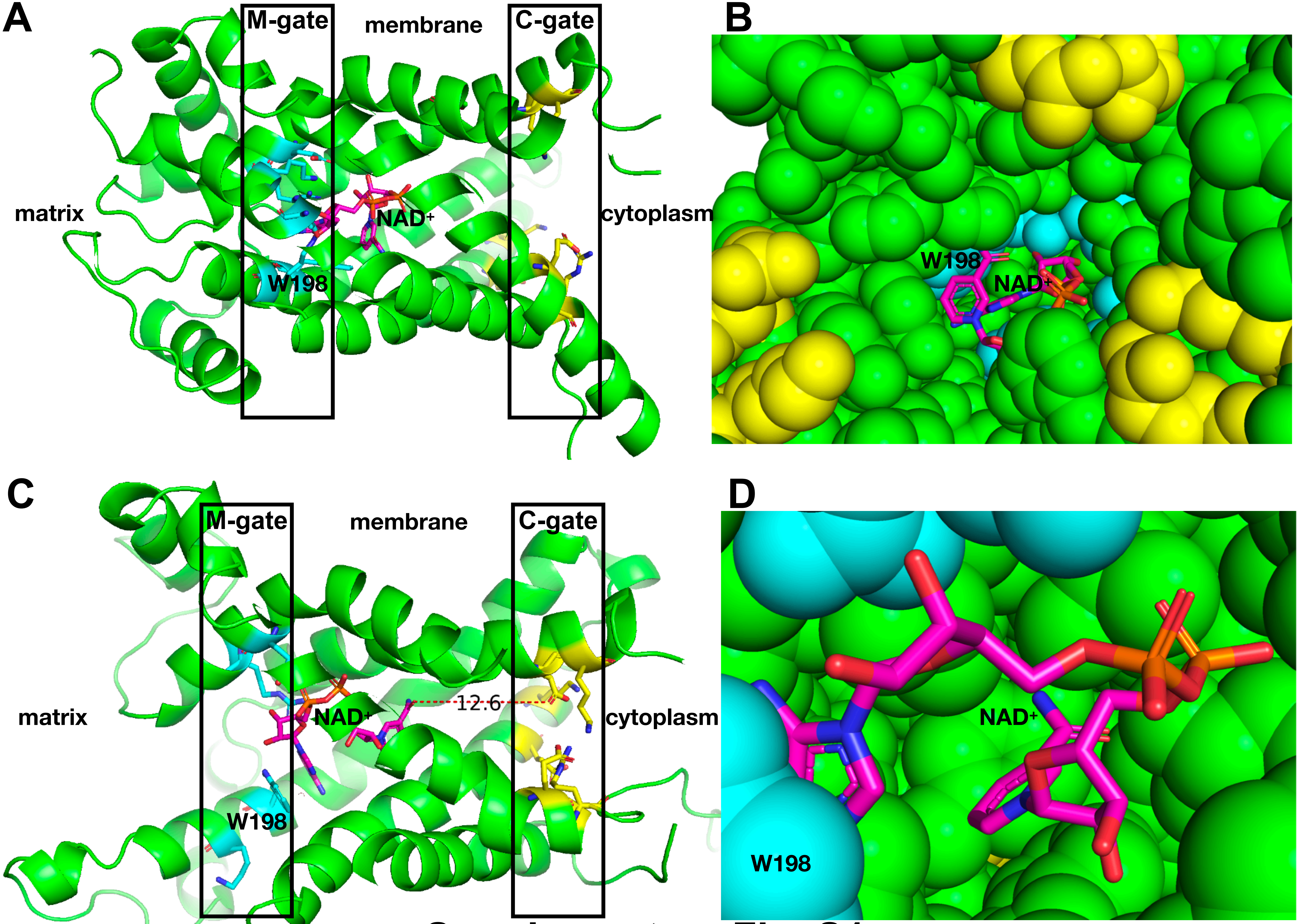

Supplementary Fig. S1

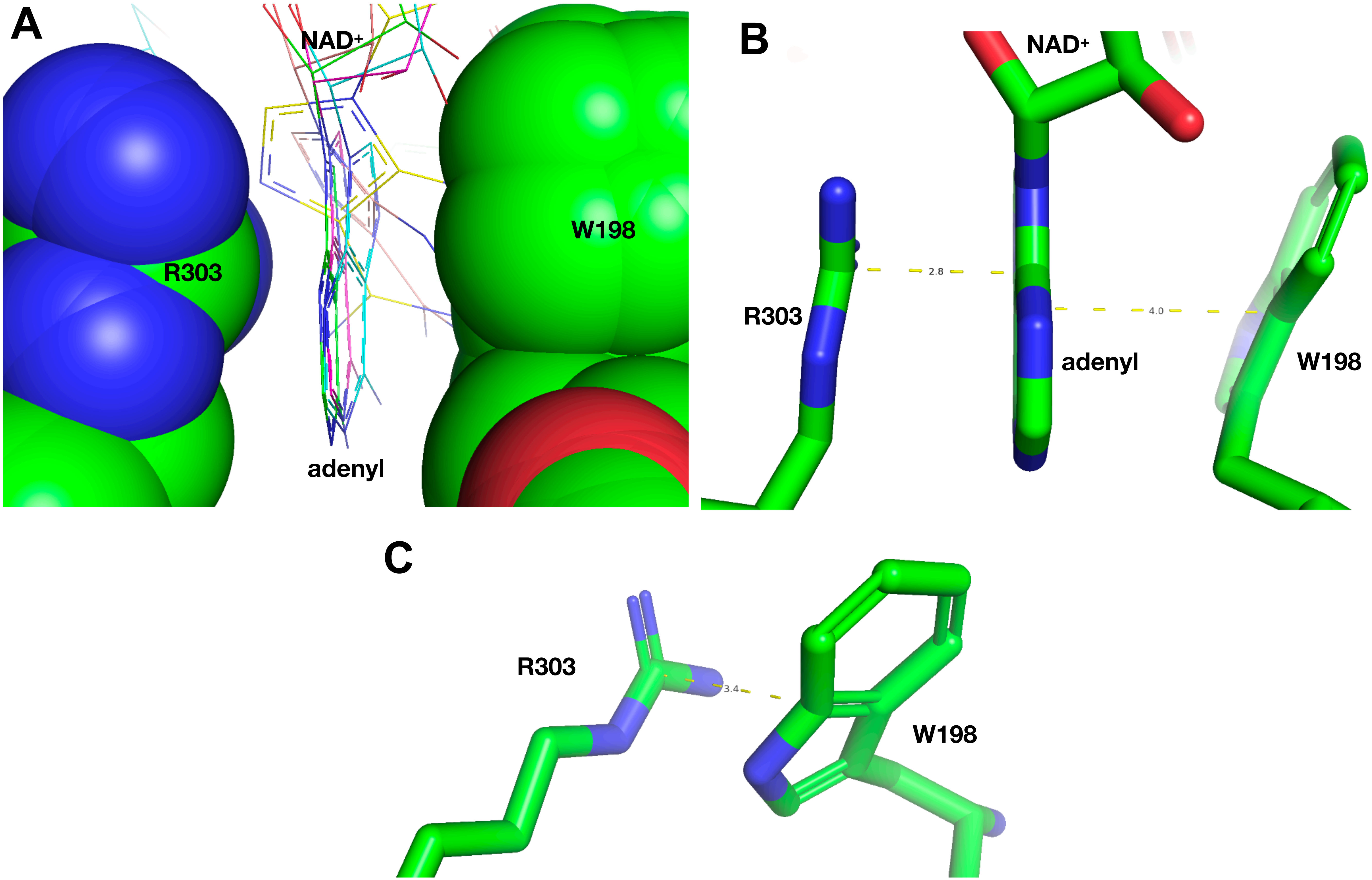

Supplementary Fig. S2

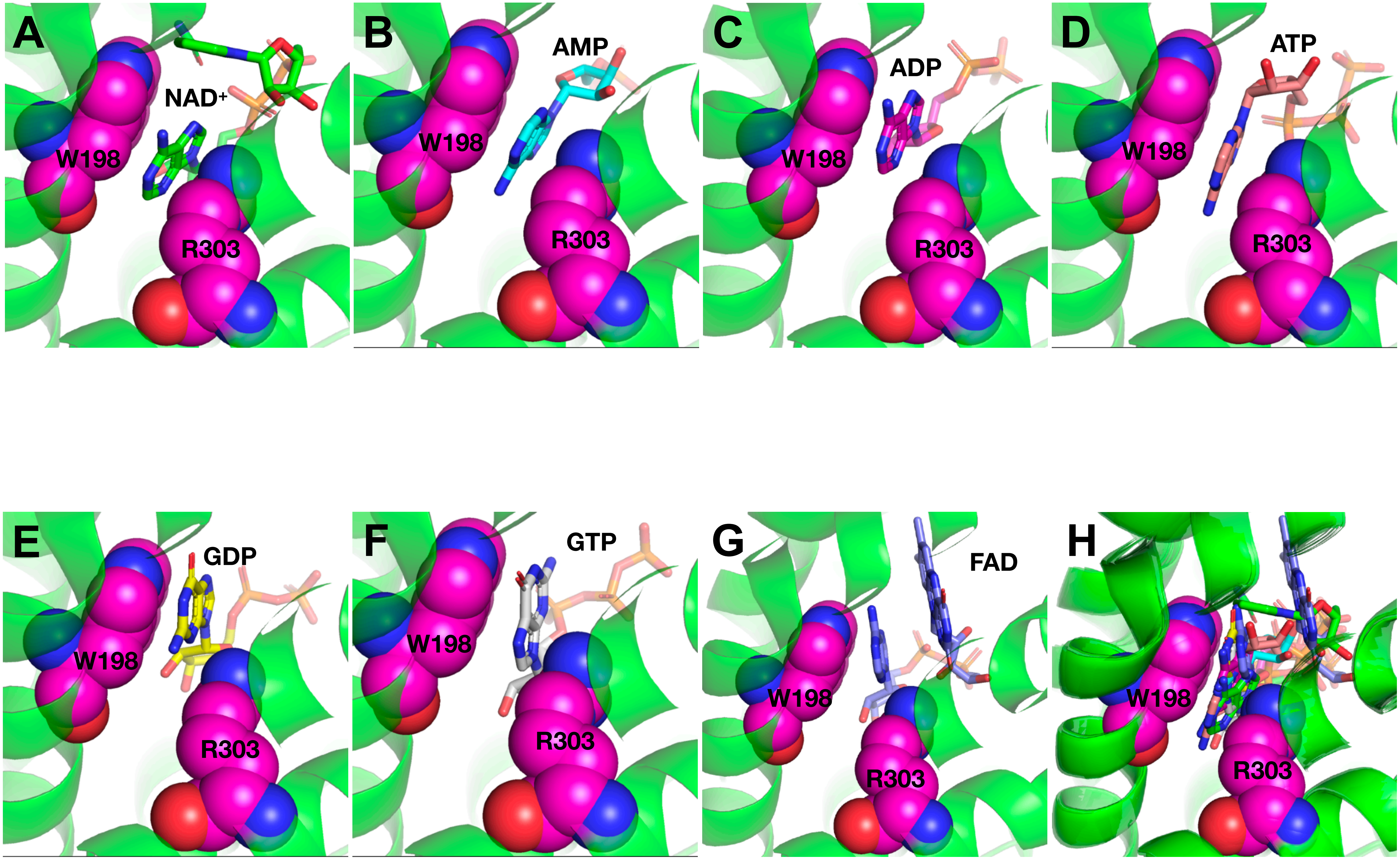

Supplementary Fig. S3

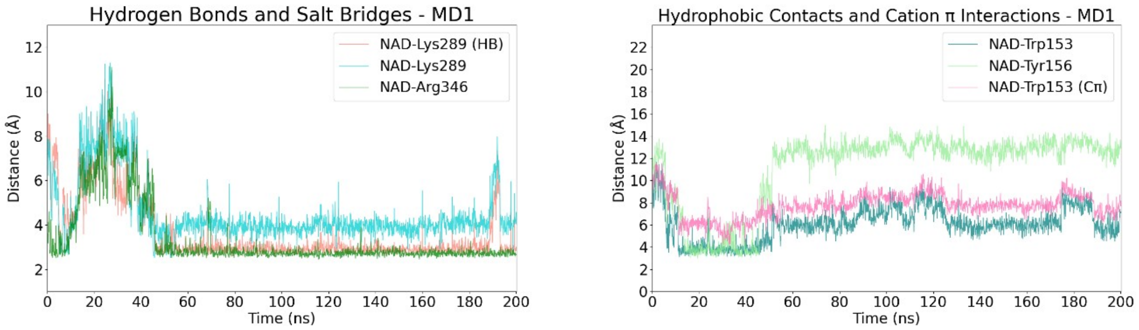

Supplementary Fig. S4. Distances between NAD^+ and binding residues during MD1.

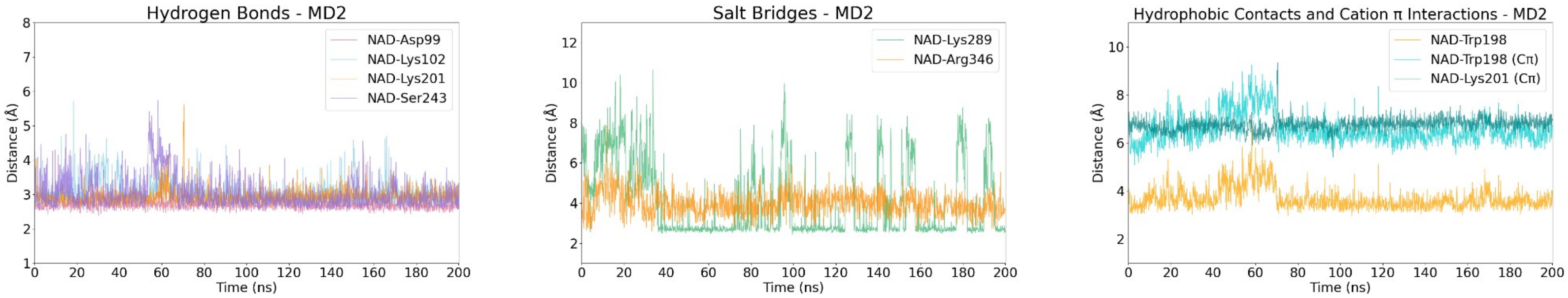

Supplementary Fig. S5. Distances between NAD^{+} and binding residues during MD2.

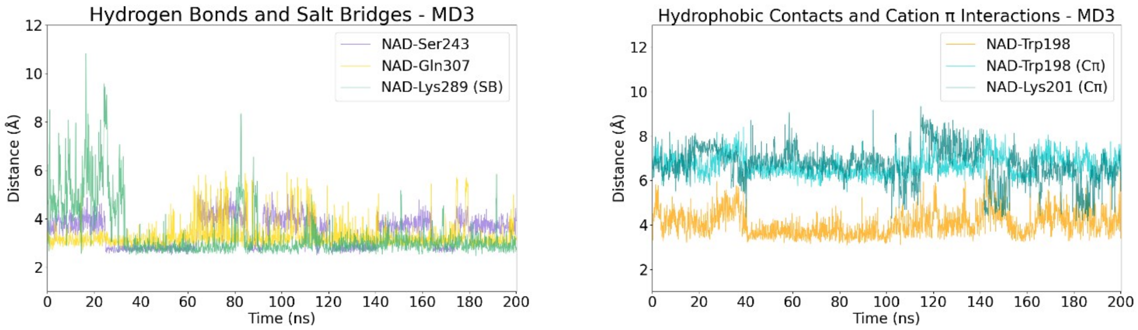

Supplementary Fig. S6. Distances between NAD⁺ and binding residues during MD3.

The PX[DE]XX[KR] signature motifs in the repeats and transmembrane helices:

		**Second repeat in H3**				**Third repeat in H5**	
Bovine AAC1	126	TSLCFVYY**P**L**D**FA**R**TRLAADVGK	147	…	224	AGLVSY**P**F**D**TV**R**RRMMMQ	241
S. cerevisiae Ndt1p	189	ASTTLTN**P**I**W**VV**K**TRLMLQSNL	210	…	292	ASAVTY**P**H**E**IL**R**TRMQLK	309
S. cerevisiae Ndt2p	151	ISTVATN**P**I**W**VV**K**TRLMLQTGI	172	…	257	ASTVTY**P**H**E**IL**R**TRMQLK	274
S. cerevisiae Rim2p	188	ATATATN**P**I**W**LI**K**TRVQLDKAG	209	…	302	ASIATY**P**H**E**VV**R**TRLRQT	319
A. thaliana PXN	124	VNVLMTN**P**I**W**VI**V**TRMQTHRKM	145	…	245	ATVTTY**P**L**L**VV**K**SRLQAK	262
A. thaliana NDT1	126	ATTIATN**P**L**W**VV**K**TRLQTQ-GM	146	…	227	ASTLTY**P**H**E**VV**R**ARLQEQ	244
A. thaliana NDT2	130	ATSIATN**P**L**W**VV**K**TRLMTQ-GI	150	…	231	ASILTY**P**H**E**VI**R**AKLQEQ	248
H. sapiens SLC25A17	114	VNVLLTT**P**L**W**VV**N**TRLKLQGAK	135	…	216	ATTVTY**P**L**Q**TV**Q**SILRFG	233
H. sapiens SLC25A32	133	MTLCITN**P**L**W**VT**K**TRLMLQYDA	154	…	238	AVAATY**P**Y**Q**VV**R**ARLQDQ	255
H. sapiens SLC25A33	141	ITNSLMN**P**I**W**MV**K**TRMQLEQKV	162	…	247	ASCIAY**P**H**E**VI**R**TRLREE	264
H. sapiens SLC25A36	131	TAITATN**P**I**W**LI**K**TRLQLDARN	152	…	240	ATTIAY**P**H**E**VV**R**TRLREE	257

Supplementary Fig. S7.

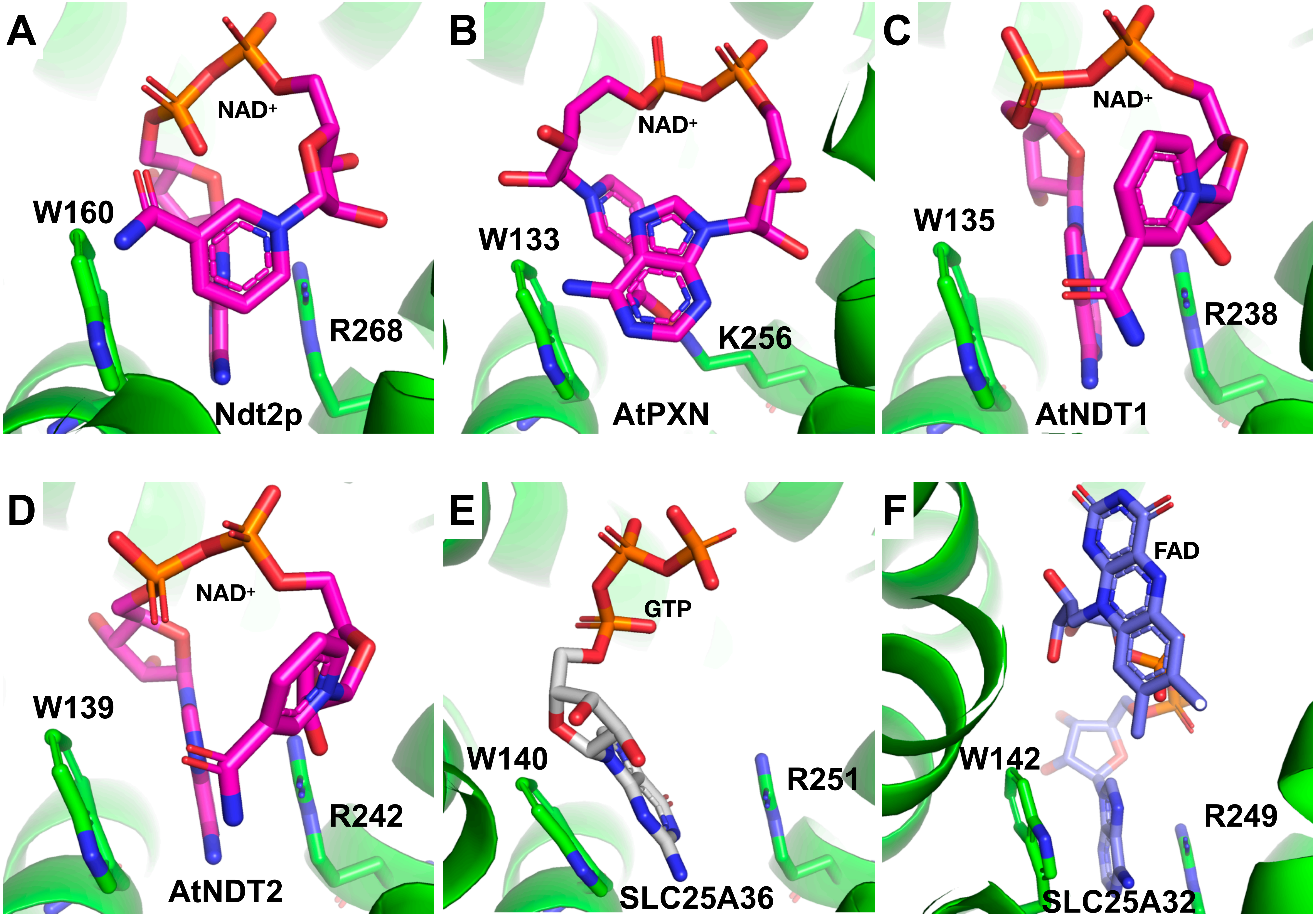

Supplementary Fig. S8

E161C for	tcc gtg tat TGC ttc agc aaa
E161C rev	ttt gct gaa GCA ata cac gga
K164C for	gaa ttc agc TGC aag ttc ttt
K164C rev	aaa gaa ctt GCA gct gaa ttc
E258C for	cct ata tac TGC gat ttg aag
E258C rev	ctt caa atc GCA gta tat agg
K261C for	gaa gat ttg TGC gta aga ttt
K261C rev	aaa tct tac GCA caa atc ttc
E359C for	gtg tcc ttt TGC tat ttc aga
E359C rev	tct gaa ata GCA aaa gga cac
R362C for	gag tat ttc TGC aac cgc cta
R362C rev	tag gcg gtt GCA gaa ata ctc

E161R for	tcc gtg tat CGA ttc agc
E161R rev	gct gaa TCG ata cac gga
K164E for	ttc agc GAA aag ttc ttt
K164E rev	aaa gaa ctt TTC gct gaa
E258K for	cct ata tac AAA gat ttg
E258K rev	caa atc TTT gta tat agg
K261E for	gat ttg GAA gta aga ttt
K261E rev	aaa tct tac TTC caa atc
E359K for	gtg tcc ttt AAA tat ttc
E359K rev	gaa ata TTT aaa gga cac
R362E for	tat ttc GAA aac cgc cta
R362E rev	tag gcg gtt TTC gaa ata

Supplementary Fig. S9

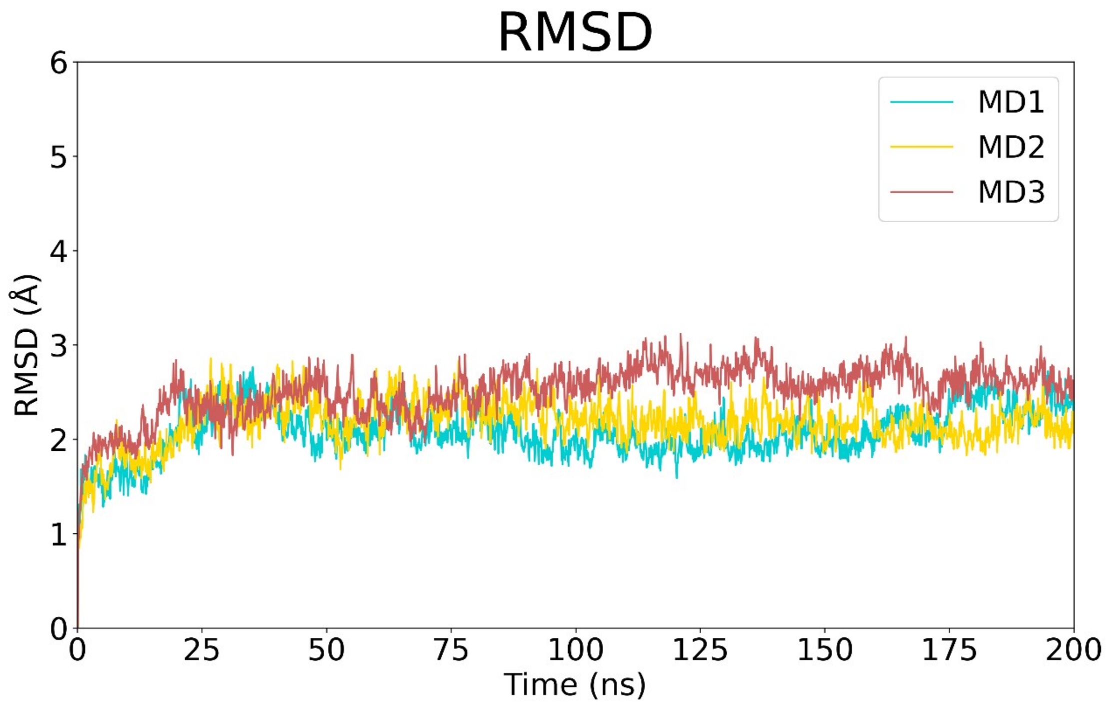

Supplementary Fig. S10. RMSD time series. Plots of the RMSD time series for each simulation, calculated for the protein backbone from residue 1 to residue 305, which correspond to residues 69-373 of the wild-type sequence.

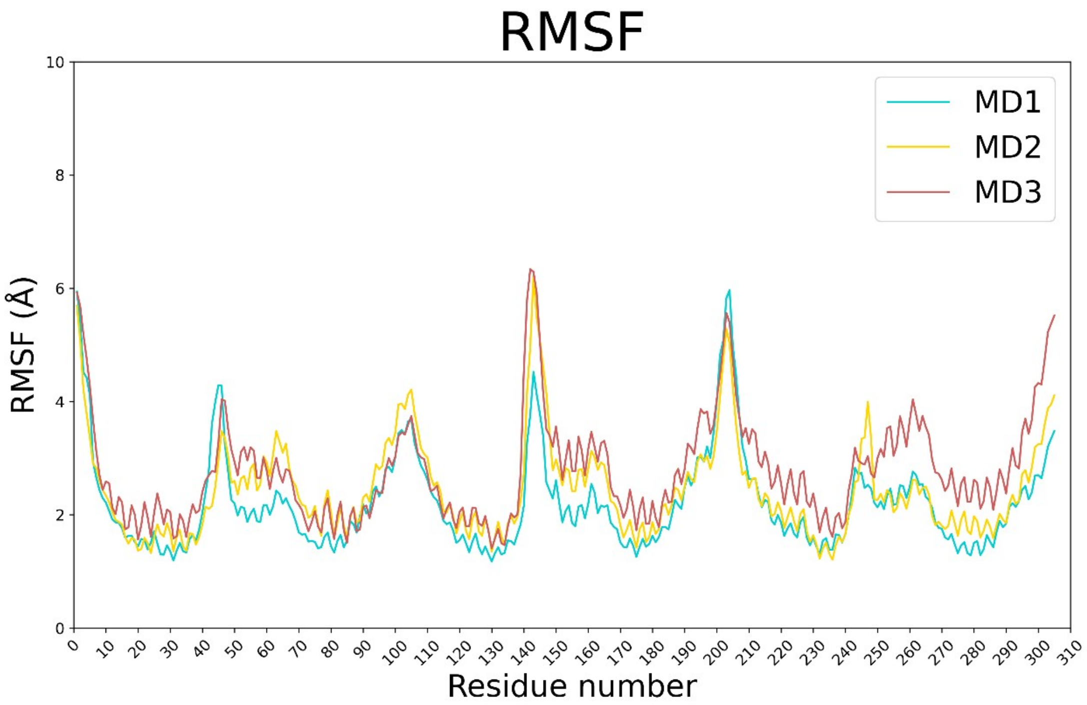

Supplementary Fig. S11. RMSF analysis. Plots of the RMSF for each simulation calculated from residue 1 to 305, which correspond to residues 69-373 of the wild-type sequence.

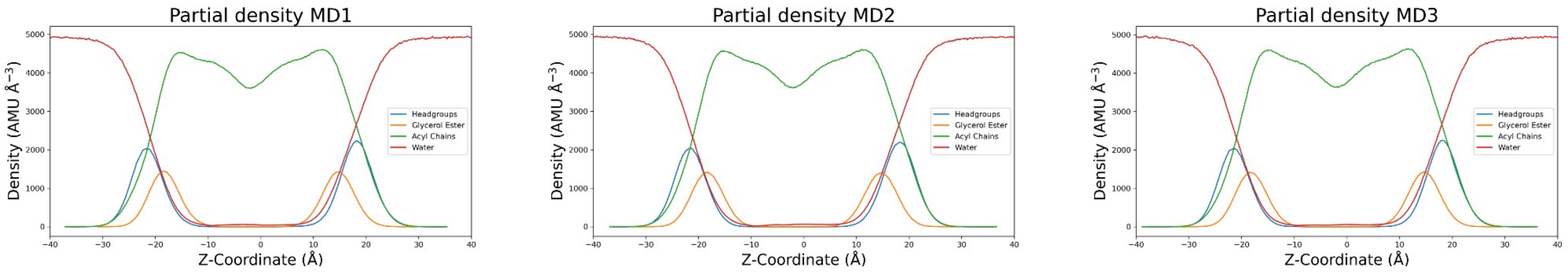

Supplementary Fig. S12. Mass density calculated for lipids headgroups, glycerol ester groups, acyl chains, and for the solvent, during each simulation.

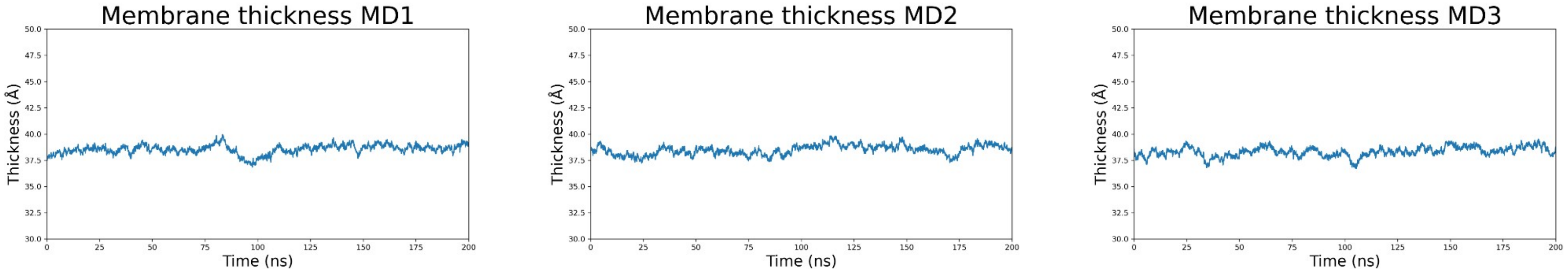

Supplementary Fig. S13. Bilayer lipid membrane thickness time series. Thickness of the bilayer lipid membrane is plotted against simulation time.

Supplementary Table S1. Interactions established by NAD^+ during the first MD simulation. For each pair, only the most frequent interaction is reported and only those with a persistence higher than 10% of the trajectory are shown. For cation-π interactions only the most frequent interaction is reported but the evaluation of the interaction has been carried out considering the distance between the geometric center of the aromatic ring and the positively charged group, as well as the angle formed between the cation and the aromatic ring plane.

MD1		
Hydrogen Bonds		
Acceptor	Donor	Fraction
NAD@O5D	LYS_289@NZ	0.180
Salt Brigdes		
Acceptor	Donor	Fraction
NAD@O2A	ARG_346@NH2	0.820
NAD@O2N	LYS_289@NZ	0.481
Hydrophobic Contacts		
Contact		Fraction
NAD@C4N : TRP_153@CZ2		0.132
NAD@C5N : TYR_156@CZ		0.111
**Cation-π Interactions**		
NAD@N1N : TRP_153		0.309

Supplementary Table S2. Interactions established by NAD^+ during the second MD simulation. For each pair, only the most frequent interaction is reported and only those with a persistence higher than 10% of the trajectory are shown. For cation-π interactions only the most frequent interaction is reported but the evaluation of the interaction has been carried out considering the distance between the geometric center of the aromatic ring and the positively charged group, as well as the angle formed between the cation and the aromatic ring plane. The abbreviation ADE indicates the adenosine group of NAD^+, whose ring is involved in cation-π interactions.

	MD2	
	Hydrogen Bonds	
Acceptor	Donor	Fraction
ASP_99@OD2	NAD@O3B	0.955
SER_243@O	NAD@O2D	0.575
NAD@O2B	LYS_201@NZ	0.270
NAD@O4B	LYS_102@NZ	0.162
	Salt Brigdes	
Acceptor	Donor	Fraction
NAD_306@O1N	LYS_289@NZ	0.618
NAD_306@O2A	ARG_346@NH1	0.534
	Hydrophobic Contacts	
Contact		Fraction
NAD@C4N : TRP_198@CE2		0.823
	**Cation-π Interactions**	
LYS_201@NZ : NAD@ADE		0.92
NAD@N1N : TRP_198		0.382

Supplementary Table S3. Interactions established by NAD+ during the third MD simulation. For each pair, only the most frequent interaction is reported and only those with a persistence higher than 10% of the trajectory are shown. For cation-π interactions only the most frequent interaction is reported but the evaluation of the interaction has been carried out considering the distance between the geometric center of the aromatic ring and the positively charged group, as well as the angle formed between the cation and the aromatic ring plane. The abbreviation ADE indicates the adenosine group of NAD+, whose ring is involved in cation-π interactions.

	MD3	
	Hydrogen Bonds	
Acceptor	Donor	Fraction
NAD@O2D	SER_243@OG	0.358
NAD@N1A	GLN_307@NE2	0.221
	Salt Brigdes	
Acceptor	Donor	Fraction
NAD_306@O1N	LYS_289@NZ	0.812
	Hydrophobic Contacts	
Contact		Fraction
NAD@C4N_198@CE2		0.513
	**Cation-π Interactions**	
LYS_201@NZ : NAD@ADE		0.715
LYS_102@NZ : NAD@ADE		0.349
NAD@N1N : TRP_198		0.278

Supplementary Table S4. Membrane composition for MD simulations. During membrane building with CHARMM-GUI the following number of lipids and mean area per lipid (APL) have been selected.

Lipid	Number in the outer leaflet	Number in the inner leaflet	Mean APL (Å)
POPE	5	4	63.6
POPC	10	3	64.03
SAPC	32	17	64.995
POPE	5	4	62.675
PLPE	8	8	62.99
SAPE	24	24	64.115
SAPI	5	6	64.525
SAPS	3	3	64.085
TLCL1	11	25	133.775
